# Identification of the Ligands of TCRγδ by Screening the Immune Repertoire of γδT Cells From Patients With Tuberculosis

**DOI:** 10.3389/fimmu.2019.02282

**Published:** 2019-09-24

**Authors:** Yuxia Li, Xinfeng Wang, Da Teng, Hui Chen, Maoshui Wang, Junling Wang, Jianmin Zhang, Wei He

**Affiliations:** ^1^State Key Laboratory of Medical Molecular Biology, Department of Immunology, Research Center on Pediatric Development and Diseases, Institute of Basic Medical Sciences, Peking Union Medical College, Chinese Academy of Medical Sciences and School of Basic Medicine, Beijing, China; ^2^Department of Laboratory Medicine, Shandong Provincial Chest Hospital, Jinan, China

**Keywords:** *Mycobacterium tuberculosis*, γδT cells, immune repertoire, tuberculosis, ligands

## Abstract

Tuberculosis (TB) caused by *Mycobacterium tuberculosis* (*Mtb*) infection is a serious threat to human health. γδT cells, which are characterized by major histocompatibility complex (MHC) non-restriction, are rapidly activated and initiate anti-infectious immune responses in the early stages of *Mtb* infection. However, the mechanism underlying the recognition of *Mtb* by γδT cells remains unclear. In this study, we characterized the pattern of the human T-cell receptor (TCR) γδ complementary determinant region 3 (CDR3) repertoire in TB patients by using high-throughput immune repertoire sequencing. The results showed that the diversity of CDR3δ was significantly reduced and that the frequency of different gene fragments (*V*/*J*), particularly the *V*-segment of the δ-chain, was substantially altered, which indicate that TB infection-related γδT cells, especially the δ genes, were activated and amplified in TB patients. Then, we screened the *Mtb*-associated epitopes/proteins recognized by γδT cells using an *Mtb* proteome chip with dominant CDR3δ peptides as probes. We identified the *Mtb* protein Rv0002 as a potential ligand capable of stimulating the activation and proliferation of γδT cells. Our findings provide a further understanding of the mechanisms underlying γδT cell-mediated immunity against *Mtb* infection.

## Introduction

Tuberculosis (TB) caused by *Mycobacterium tuberculosis* (*Mtb*) infection is a serious threat to human health. According to the Global Tuberculosis Report 2018, worldwide, TB is one of the top 10 causes of death and the leading cause from a single infectious agent. An estimated 10.0 million people fell ill with TB in 2017, and 90% of these people were adults (1). However, TB is a chronic infectious disease with persistent pathogens. Conventional antituberculosis drugs sometimes fail to yield good results due to the high rate of drug resistance and the proliferation of multiple drug-resistant TB strains. Globally, ~558,000 new patients in 2017 were resistant to rifampicin, while 82% had multidrug-resistant tuberculosis (MDR-TB) (1). Therefore, TB is still a worldwide public health problem that must be solved.

Unfortunately, a clear picture of the interactions between *Mtb* and the immune system has yet to emerge. Studies have shown that T cells such as CD8^+^ T cells, CD4^+^ T cells, NK cells, γδT cells, and CD1-restricted T cells play important roles against TB infection (2). On the one hand, *Mtb* not only inhibits apoptosis but also prevents dendritic cells from presenting antigens, leading to a delay in the early T cell response to *Mtb* (3). Moreover, *Mtb* can induce the expansion of TB-specific regulatory T cells, thereby delaying the immune response (4, 5). As an innate immune cell type, γδT cells represent only 1~5% of human peripheral blood lymphocytes, but they predominate in tissues such as the skin, tongue mucosa, and respiratory epithelium. However, first contact between *Mtb* and the body occurs precisely in the respiratory epithelial mucosa and alveolar surface. Early studies have shown that the number of human Vγ9^+^/Vδ2^+^ T cells is reduced in the peripheral blood of patients with active pulmonary TB, indicating that Vγ9^+^/Vδ2^+^T cells play a protective role (6). Many studies have investigated the activation, proliferation, apoptosis and mechanisms of γδT cells during *Mtb* infection. Previous results have confirmed that γδT cells can provide protection to the host in the early stages of infection (7–10).

Changes in the T cell receptor (TCR) repertoire can reflect the state of the human immune system. Therefore, the characteristics of the TCR repertoire have been widely studied in many diseases, such as HIV infection (11), rheumatoid arthritis (12, 13) and lung carcinoma (14). In the early stage of *Mtb* infection, γδT cells recognize *Mtb*-associated antigens through the diverse TCRγδ repertoire (15). Therefore, the receptor repertoire will certainly change. Similar to the CDR3 region of the heavy chain of an antibody, CDR3δ is particularly important in TCRγδ antigen recognition (16). We previously demonstrated that the primary structure of CDR3δ can determine the antigen-binding activity of the entire TCRγδ (17). This observation suggests that changes in the CDR3δ repertoire can reflect *Mtb* infection. Therefore, studying the correlation between the CDR3δ repertoire and TB will identify the specific CDR3δ sequence profile and provide new clues for understanding the mechanisms of γδT cells in the defense against Mtb infection.

γδT cells can be stimulated by *Mtb* to produce interferon-γ (IFN-γ) and interleukin 17 (IL-17), which mediate the immune response caused by acute and chronic infection and protect the human body (18). IFN-γ is significantly reduced while IL-17 is significantly increased in TB patients compared with in bacillus calmette-guérin (BCG)-stimulated healthy controls (19). Previous studies in our laboratory showed that the recognition of some protein ligands by γδT cells is based on the dual recognition mechanism of both TCRγδ and natural killer group 2 member D (NKG2D) (20). However, the mechanism of *Mtb* recognition by γδT cells remains unclear. Currently, studies of TCRγδ ligands have focused on non-peptide phosphorylation antigens represented by isoprene pyrophosphate (IPP). Further elucidating the anti-*Mtb* infection mechanism of γδT cells therefore requires identifying additional *Mtb*-specific TCRγδ ligands.

In this study, we analyzed the characteristics of the TCRγδ CDR3 repertoire with respect to *Mtb* infection in TB patients and BCG vaccine stimulation *in vitro* using high-throughput sequencing and identified 10 specific CDR3δ dominant sequences related to *Mtb* infection. In addition, we used *Mtb*-specific CDR3δ sequences as probes to screen for epitopes/proteins. Finally, we identified the *Mtb* protein Rv0002 as a novel ligand for TCRγδ. This protein can stimulate γδT cell activation in the peripheral blood of healthy controls and TB patients and can stimulate γδT cell proliferation in TB patients. This study provides a molecular basis for γδT cell-mediated resistance to *Mtb* infection and for the development of anti-tuberculosis research.

## Materials and Methods

### Study Subjects

Peripheral blood samples for phenotypic analysis and high-throughput sequencing were obtained from 14 patients (age, 40.1 ± 19.5 years; the ratio of male to female was 11:3), including 10 cases with secondary pulmonary tuberculosis, 3 cases with tuberculous pleurisy and one with cavity tuberculosis. All patients were newly diagnosed with acute TB infection and had not been treated with anti-tuberculosis drugs. The characteristics of these patients are summarized in Table S1. Fifteen healthy volunteers (age, 44.6 ± 10.9 years; male: female ratio, 7:8) were included as healthy controls (HCs). All patients and healthy volunteers signed donation consent forms before sample collection. None of the healthy volunteers had a history of tuberculosis or any other underlying disease including viral and other bacterial infections/co-infections, severe hepatic diseases, renal diseases, immunological, or autoimmune diseases. This study was carried out in accordance with the recommendations of the Ethical Committee of the Chinese Academy of Medical Sciences with written informed consent from all subjects. All subjects gave written informed consent in accordance with the Declaration of Helsinki. The protocol was approved by the Ethical Committee of the Chinese Academy of Medical Sciences. Samples for high-throughput sequencing were frozen in RNA protection reagent (Qiagen, Hilden, Germany) for further processing.

### Peripheral Blood Collection and Isolation of Mononuclear Lymphocytes

All peripheral blood samples were collected in aspiration vessels containing ethylene diamine tetraacetic acid (EDTA) anticoagulant. Peripheral blood mononuclear cells (PBMCs) were freshly isolated by density-gradient centrifugation using human lymphocyte separation medium. γδT cells were cultured from PBMCs in RPMI-1640 medium (Gibco, CA, USA) supplemented with 10% fetal calf serum (FCS, Gibco, CA, USA), penicillin, streptomycin, and 200 IU/mL of IL-2 in a 24-well culture plate containing immobilized anti-TCR PAN γ/δ monoclonal antibody (Beckman Coulter, CA, USA) at 37°C in 5% CO_2_. After 96 h, the cells were transferred to a new 24-well plate, and half of the medium was discarded and replaced with fresh medium and cytokines. After 10 days of culture, the purities of γδT cells were assessed by flow cytometry and over 85% purity was obtained before the cultures were used for further experiments.

### Screening for CDR3δ Peptide-Binding Phage Clones in the Ph.D.-12^TM^ Phage Display Peptide Library

The Ph.D.-12^TM^ Phage Display Peptide Library (New England Biolabs, MA, USA) was screened using CDR3δ peptides as follows: a 96-well plate was coated overnight with 150 μL/well of coating buffer (containing 10 μg of CDR3δ peptide) at 4°C and then blocked overnight with 0.1 M NaHCO_3_ (pH 8.6) containing 5 mg/mL bovine serum albumin at 4°C. The primary library solution was added to the wells (10 μL per well containing 10^11^ colony-forming units) and then shaken gently at room temperature for 30 min. After thoroughly washing with Tris-buffered saline (TBS) containing 0.1% Tween 20, the CDR3δ peptide-binding phages were eluted in acidic buffer [0.2 M glycine-HCl (pH 2.2)], followed by immediate neutralization with 1 M Tris-HCl (pH 9.1) or by competitive elution with a high concentration of CDR3δ peptide (100 μg/mL). The concentration of Tween 20 in the washing buffer was 0.1% in the first round and 0.5% in the subsequent two rounds of screening.

### Identification of CDR3δ Peptide-Binding Phage Clones by Phage-ELISA, DNA Extraction, and Sequencing

After three rounds of screening, each set of phage clones that was amplified was detected by phage enzyme-linked immunosorbent assay (ELISA) using a horseradish peroxidase (HRP)-conjugated anti-M13 phage antibody (GE Healthcare). Briefly, the wells of the ELISA plate were coated with CDR3δ peptides and blocked as above. Amplified phage clones were added to the CDR3δ peptide-coated wells (10 μL per well containing 10^11^ colony-forming units) and incubated for 2 h at room temperature with agitation. After thorough washing, HRP-anti-M13 phage antibody (1:5,000 dilution) was added and incubated for 1 h at room temperature with agitation. The final set of phage clones that was enriched was collected and subjected to DNA extraction. Briefly, 20% PEG/2.5 M NaCl was added to the phage stock and incubated at room temperature for 20 min. Samples were centrifuged at 12,000 × g for 10 min at 4°C. Phage pellets were thoroughly resuspended in iodide buffer, 250 μL of ethanol was added, and the samples were incubated for 20 min at room temperature. The samples were centrifuged again (10 min at 12,000 × g, 4°C), and the pellets were washed with 0.5 mL of 70% ethanol, respun, dried briefly, and resuspended in 30 μL of TE buffer [10 mM Tris-HCl (pH 8.0), 1 mM EDTA]. The products were quantitated by agarose gel electrophoresis. The phage DNA was PCR-amplified using the following specific primers: 5′-TTATTCGCAATTCCTTTAGTG-3′; 5′-GCCCTCATAGTTAGCGTAACG-3′. The PCR products were purified using a PCR product purification kit (Qiagen, Hilden, Germany) and sequenced via high-throughput sequencing (GENEWIZ, New Jersey, USA).

### Analysis of Sequencing Data

The raw data were deposited in the Genome Sequence Archive (Genomics, Proteomics and Bioinformatics 2017) in BIG Data Center (Nucleic Acids Res 2018), Beijing Institute of Genomics (BIG), Chinese Academy of Sciences, under accession number CRA001614 that are publicly accessible at http://bigd.big.ac.cn/gsa.

Raw data were analyzed by iRepertoire using the IRmap program to identify CDR3s for each sample. The best matches of the germline *V* and *J* genes were identified by determining alignments between the Illumina platform product and germline sequences in the IMGT/GENE-DB database. All of the TCRγδ CDR3 repertoire analyses in this study were limited to in-frame sequences. We used the diversity 50 (D50) value, the unique CDR3/total CDR3, the CDR3 clonal size and the frequency of dominant sequences to comprehensively analyze differences between TB patients and HCs in terms of CDR3 diversity. The total CDR3 sequences referred to all of the CDR3 sequences obtained from the filtered sequences, and the unique CDR3 sequences referred to each distinct CDR3 sequence regardless of how many copies appeared. The D50 value represents the number of unique CDR3 sequences contained in total CDR3 sequences when the frequency reaches 50% of the number of total CDR3s sorted from high to low frequency. The percentages of each germline *V* and *J* gene segment were plotted to easily identify the frequently and infrequently used *V* and *J* alleles based on normalized data. The number of CDR3 nucleotides and random insert (Naddition) for each unique CDR3 (normalized data) were recorded, and related distribution plots were prepared in accordance with the proportion to demonstrate the distribution of the entire CDR3 repertoire. Based on the length and proportion, we calculated the weighted average to evaluate the differences in CDR3 and N-addition length distribution. The CDR3 immune repertoire characteristics of γδT cells before and after stimulation by BCG were also analyzed using the above methods.

### Screening Proteins Bound to CDR3δ Peptides Using an *Mtb* Proteome Chip

*Mtb*-proteins were screened using CDR3δ peptides with an *Mtb* proteome chip as follows: The chip was brought to room temperature, blocked with 3% bovine serum albumin-phosphate buffered saline Tween 20 (0.1%) (BSA-PBST), and gently shaken for 2 h at room temperature. The chip was then incubated with the peptide solution (1 μg/mL in 3% BSA-PBST) and shaken gently for 2 h at room temperature. After 3 washes with PBST, the chip was incubated with Cy3 streptavidin (Biolegend, CA, USA) (1:500 in 3% BSA-PBST) and shaken gently at room temperature for 1 h in the dark. The chip was washed as above and incubated for 1 h with GST rabbit mAb (Cell Signaling Technology, MA, USA) (1:500 in 3% BSA-PBST) in the dark with gentle shaking. After washing, the chip was incubated for 1 h with IgG Fab Alexa Fluor 647 (Cell Signaling Technology, MA, USA) (1:800 in 3% BSA-PBST) in the dark with gentle shaking. The chip was thoroughly washed and stored in the dark at 4°C.

### ELISA

Binding between the epitope peptides and probe: ELISA plates were coated overnight with probe (20 μg) at 4°C and then blocked with 5% BSA-PBST for 2 h at 37°C. Different concentrations of biotin-conjugated epitope peptides (Biotin-EPs) and controls were co-cultured with the probes for 2 h at 37°C. The plates were washed 6 times with 0.05% PBST and then incubated with HRP-conjugated streptavidin (1:3,000 in 0.05% PBST) for 30 min at 37°C. The absorbance at 450 nm was used to determine binding. Binding between Rv0002 and the probe: ELISA plates were coated overnight with different doses of Rv0002 at 4°C and then blocked for 2 h with 5% skim milk (in PBST) at 37°C. Next, 100 μg/mL of probes were cocultured with Rv0002 for 2 h at 37°C. The plates were washed 6 times with 0.05% PBST and then incubated with HRP-conjugated streptavidin (1:500 in 0.05% PBST) for 30 min at 37°C. The absorbance at 450 nm was used to determine binding.

### PBMC Culture With BCG Vaccine/Rv0002 Proteins

For BCG vaccine stimulation, PBMCs were freshly isolated and cultured with BCG at 10 and 20 μg/mL for 72 h. Then, the cells were transferred to a new 48-well plate, and part of the cells were collected for flow cytometry to detect the proportion of γδT cells and the expression of CD25 and CD69. Every other day, the cells were tested until the 15th day of culture. We collected three pairs of PBMCs before and after stimulation with 20 μg of BCG, and stored them with RNA protection reagent for high-throughput sequencing. For Rv0002 protein stimulation, Rv0002 proteins (20 μg/mL) were immobilized on the plates to stimulate the PBMCs from HC and TB patients. The culture and detections were performed as BCG vaccine stimulation.

### Proliferation Assay

The proliferation of γδT cells was detected by CellTrace™ CFSE Cell Proliferation Kit (Invitrogen™, Thermo Fisher, USA) as described previously (21, 22). In brief, γδT cells were amplified *in vitro* with anti-TCR PAN γ/δ monoclonal antibody (Beckman Coulter, CA, USA). When the purity of γδT cells was >85%, IL-2 was removed from the medium and the cells were allowed to stand for 48 h. The cells were collected and washed with RPMI-1640 basal medium to remove the FCS and resuspended in 1 × PBS containing 0.1% FCS to a concentration of 1 × 10^6^/mL. Then, CFSE was added to the cell suspension at a final concentration of 0.5 μmol/L, and the cells were incubated for 10–20 min at 37°C in 5% CO_2_. Five volumes of precooled CFSE stop solution (10% FBS RPMI-1640) were immediately added to the tubes and placed at 37°C for 5 min. After centrifugation, the cells were washed twice with RPMI-1640 complete medium without IL-2 and suspended in RPMI-1640 complete medium (RPMI-1640 containing 1% penicillin-streptomycin, 10% FBS, no IL-2). The cell suspension was added to a 48-well plate that was precoated with Rv0002 protein and BSA (control) and cultured at 37°C in 5% CO_2_ for 72 h. The cells were then collected for assessing proliferation by flow cytometry.

### Flow Cytometry

Flow cytometry was performed to detect the proportions of various cells and the expression of cytokines in the peripheral blood from TB patients and HCs, as described previously (21). For cell surface staining, the cells were incubated with antibodies FITC anti-human TCRγ/δ (Clone B1), PE anti-human Vδ2 (Clone B6), PE anti-human CD27 (Clone LG. 3A10), PE anti-human CD69 (Clone FN50); and APC anti-human CD25 (Clone B96) (Biolegend, CA, USA) for 30 min at 4°C. For intracellular staining, phorbol myristate acetate (50 ng/mL) and ionomycin (1 μg/mL) were added to the cell suspension, and the cells were incubated for 2 h at 37°C in 5% CO_2_. Brefeldin A (1,000 ×) was added at 1 μL/mL to the cell suspension, and the cells were incubated for 4 h at 37°C in 5% CO_2_. After collecting the cells in an EP tube (1.5 mL), the cells were washed twice with 1% BSA. After surface staining, 0.5 mL fixation/permeabilization (eBioscience™, Invitrogen™, Thermo Fisher, USA) was added, and the cells were fixed at 4°C for 30 min in the dark. Then 1 mL of permeabilization buffer (10 ×, 1:9 dilution) was used to wash the cells. After discarding the supernatant, 1 mL permeabilization buffer (10 ×, 1:9 dilution) was added, and the cells were kept at room temperature for 10 min. The supernatant was discarded after centrifugation and the cells were incubated with PE anti-human IL-17A (Clone BL168) and APC anti-human IFN-γ (Clone 4S. B3) (Biolegend, CA, USA) antibodies for 30 min at 4°C. Flow cytometry was performed using a FACSAria^TM^II and Accuri C6 flow cytometer (BD, New Jersey, USA), and the data were analyzed using FlowJo (TreeStar, San Carlos, CA, USA) and BD Accuri C6 software (BD, New Jersey, USA).

### Peptide Synthesis, Labeling, and Protein Expression

Peptides were synthesized by the peptide synthesis facility of Sangon Biotech (Shanghai), China. The purity of the synthesized peptides was more than 85% according to high-performance liquid chromatography (HPLC) analysis. Half of the synthesized peptides were N-terminally labeled with biotin. Freshly transformed *Escherichia coli* BL21(DE3) cells harboring plasmid pDEST^TM^17 were cultured in 500 mL of LB medium containing ampicillin at 37°C. When the optical density of the cells at 600 nm (OD600) reached 0.6 to 0.8, isopropyl-β-D-thiogalactoside (IPTG; Sigma) was added to a final concentration of 0.4 mM, and the bacteria were cultured for another 4 h at 37°C. The culture medium was then harvested and centrifuged at 4,000 × g for 20 min at 4°C. After ultrasonication (200 W), the samples were centrifuged at 12,000 × g for 20 min at 4°C. The pellet was dissolved in 8 M urea and then renatured via dialysis.

### Statistical Analysis

The data were analyzed using GraphPad Prism v.6 (GraphPad Software, San Diego, CA, USA) and SPSS statistic 22.0. Images were edited using Adobe Illustrator CS6 software (Adobe, CA, USA). Statistical significance was calculated using Student's *t*-test and two-tailed Mann-Whitney test to evaluate the significant differences between the two experimental conditions. *P* < 0.05 was considered a significant difference.

## Results

### The Proportion of γδT Cells Was Significantly Decreased in the Peripheral Blood of TB Patients

**Figure 1:**
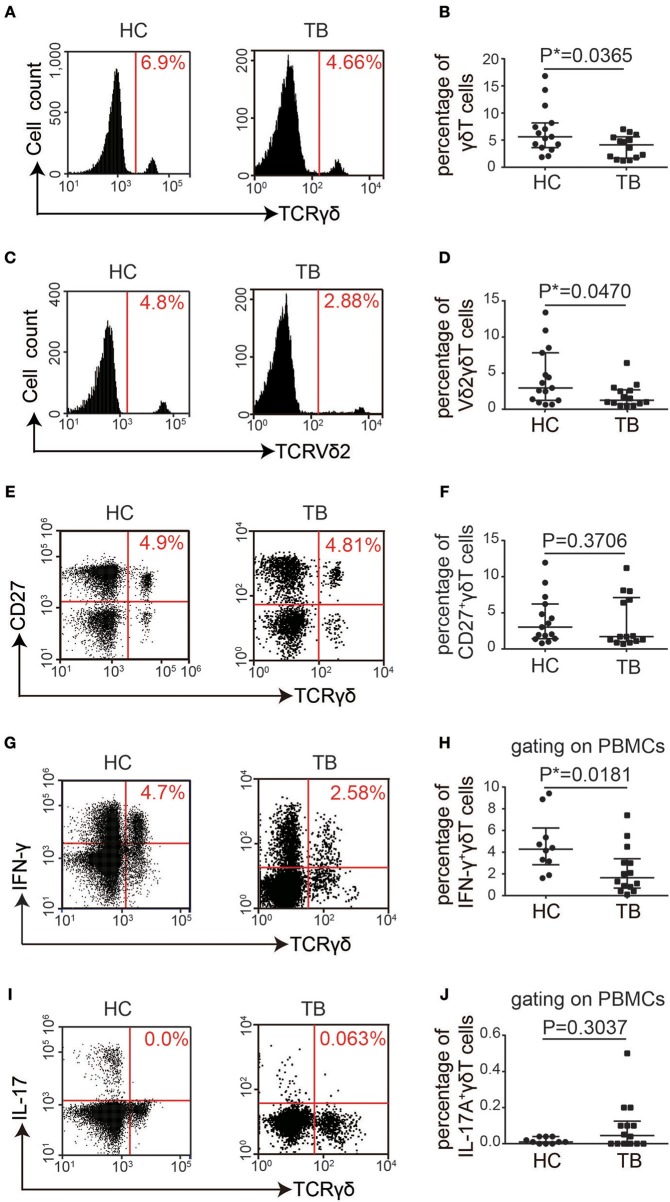
The proportion of γδT cells was significantly decreased in the PBMCs of TB patients. **(A)** Flow cytometry analysis of γδT cells in PBMCs from healthy controls (HC) and TB patients (TB). **(B)** Quantification of the proportions of total γδT cells from 15 controls and 14 TB patients. **(C,E,G,I)** Flow cytometry analysis of Vδ2γδT cells **(C)**, CD27^+^γδT cells **(E)**, IFN-γ^+^ γδT cells **(G)**, and IL-17A^+^γδT cells **(I)** in the PBMCs from 15 healthy controls and 14 TB patients. **(D,F,H,J)** Quantification of the proportions of Vδ2γδT cells **(D)**, CD27^+^γδT cells **(F)**, IFN-γ^+^ γδT cells **(H)**, and IL-17A^+^γδT cells **(J)**. Data represent the median with interquartile range. *P < 0.05 by two-tailed Mann-Whitney test.

Peripheral blood monocytes were isolated from 14 TB patients who were identified by sputum *Mtb* culture and acid-fast stain smear (Table S1). Fifteen age- and gender-matched healthy volunteers were included as controls. Flow cytometry was performed to examine the proportions of γδT cells. The proportion of γδT cells was significantly lower in the peripheral blood of TB patients than in the peripheral blood of HCs (*P* = 0.0365). This reduction in γδT cell numbers was primarily due to a decrease in Vδ2γδT cells, a major population of peripheral blood γδT cells expressing the Vδ2 chain (23) combined almost exclusively with the Vγ9 chain (24), which were referred to as Vγ9Vδ2^+^T cells. However, no significant change was observed in the proportion of CD27^+^γδT cells, a naïve, memory subset of Vγ9Vδ2^+^T lymphocytes (25, 26) (Figures 1A–F and Figures S1A–C). Next, we performed intracellular staining and flow cytometry to detect whether γδT cell cytokine secretion was altered in TB patients. The proportion of IFN-γ^+^γδT cells was significantly reduced (*P* = 0.0181), but IL-17A^+^γδT cells showed no significant difference between TB patients and HCs (*P* = 0.3037; Figures 1G–J and Figures S1D,E). Taken together, these results suggest that γδT cells, particularly Vδ2γδT cells, may be exhausted or damaged during TB infection and that this disease-related change also causes functional alterations.

### Altered Immune Repertoire of the CDR3 Region of γδT Cells in the Peripheral Blood of TB Patients

To characterize the pattern of the TCRγδ CDR3 repertoire in TB patients, PBMCs from 12 TB patients and 14 age- and sex-matched healthy controls were isolated for high-throughput immune repertoire sequencing, focusing on the CDR3 sequences of γδT cells, including the γ (TRG) and δ chains (TRD). As shown in Table S2, we analyzed six characteristics of the immune repertoire, namely, the diversity, the frequencies of the top 50 clones, the number of CDR3 nucleotides for each unique CDR3 and the distribution of the entire repertoire, the usage frequencies of germline *V* and *J* gene fragments, the N-addition, V-trim, and J-trim length polymorphism distributions and the shared CDR3 sequences.

**Figure 2:**
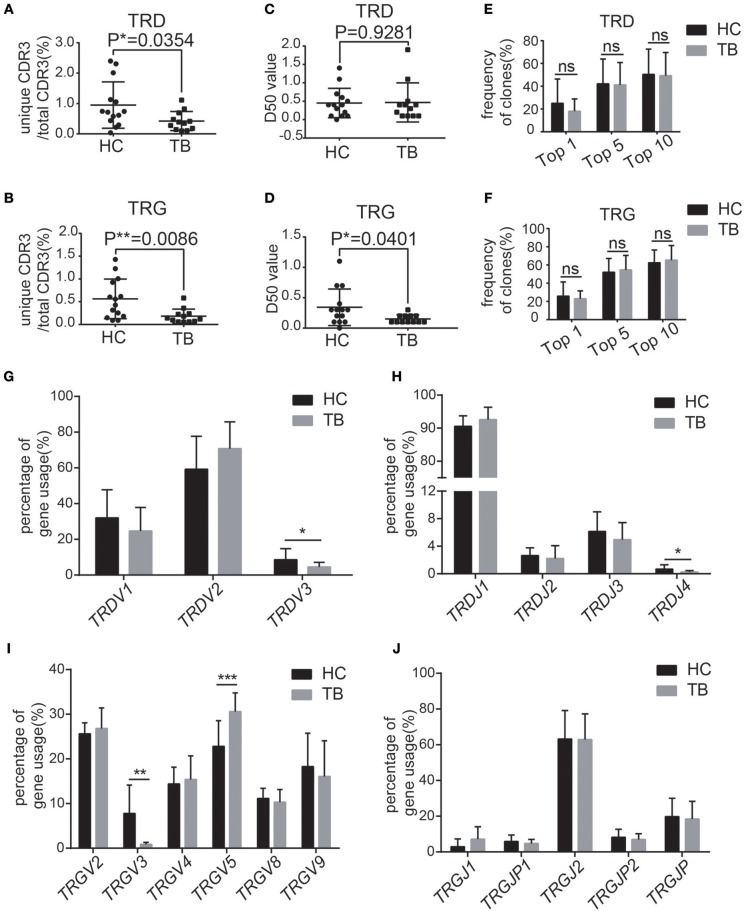
TB patients showed different characteristics of the immune repertoire of γδT cells. **(A,B)** Quantification of the ratios of unique CDR3/total CDR3 of the δ **(A)** and γ **(B)** chains in the CDR3 region. **(C,D)** Quantification of the D50 values of the δ **(C)** and γ **(D)** chains in the CDR3 region. **(E,F)** Frequencies of the top 1, top 5, and top 10 CDR3 sequences of the δ **(E)** and γ **(F)** chains in the CDR3 region. **(G,H)** The germline gene usage of T-cell receptor δ-chain (TRD) *V* fragments (*TRDV1, TRDV2, TRDV3*) and *J* fragments (*TRDJ1, TRDJ2, TRDJ3, TRDJ4*). **(I,J)** The germline gene usage of T-cell receptor γ-chain (TRG) *V* fragments (*TRGV2, TRGV3, TRGV4, TRGV5, TRGV8, TRGV9*) and J fragments (*TRGJ1, TRGJ2, TRGJP, TRGJP1, TRGJP2*). Data represent the mean ± SD. *P < 0.05, **P < 0.01, ***P < 0.001 by Student's *t* test.

We first analyzed the diversity of the immune repertoire, the most important feature of the immune repertoire that reflects the clonal proliferation of specific γδT cells. The results showed that the ratios of unique CDR3/total CDR3 of TRD and TRG were both significantly lower in TB patients than in controls (Figures 2A,B). The D50 value of TRG was significantly lower than that in controls, while TRD showed no significant difference (Figures 2C,D). The percentages of the top 1, 5, and 10 clones showed no significant differences for either TRD or TRG compared with those in the controls (Figures 2E,F). Taken together, these results demonstrate that the diversity of the CDR3 immune repertoire of γδT cells is significantly reduced in TB patients, indicating that some TB infection-related γδT cells are selectively amplified in TB patients or that certain TCR CDR3 gene fragments are preferentially utilized.

Next, we analyzed the frequencies of the top 50 clones in the repertoire (Figure S2). No significant difference in the distribution of the top 50 clones was observed for either TRD or TRG (Figures S2A–D). We also analyzed the number of CDR3 nucleotides for each unique CDR3 and the distribution of the entire repertoire. The average CDR3 lengths of TRD and TRG exhibited a standard distribution in TB patients and healthy controls, and the weighted averages of the CDR3 lengths did not significantly differ between the two groups (Figures S2E,F). These results indicate that *Mtb* infection does not alter the clone size, distribution or CDR3 length of the CDR3 region.

Next, we calculated the usage frequencies of germline *V* and *J* gene fragments in the TCRγδ CDR3 repertoire. The germline *V-J* gene usage of the TCRγδ repertoire, including three functional *V* gene segments of TRD (*hTRDV1/2/3*), six functional *V* gene segments of TRG (*TRGV2/3/4/5/8/9*), four functional *J* gene segments of TRD (*hTRDJ1/2/3/4*), and five functional *J* gene segments of TRG (*hTRGJ1/P1/2/P2/P*), were analyzed. The percentages of *hTRDV3, hTRDJ4*, and *hTRGV3* were significantly reduced while the percentages of *hTRGV5* was significantly increased in the TCRγδ CDR3 repertoire compared with the controls (Figures 2G–J). These results suggest that the usage of *V* gene fragments in both TRD and TRG was significantly altered in TB patients.

The TCRγδ CDR3 has a random insertion and deletion of the gene fragment between the *VJ* gene fragments (γ chain) and the *VD/DJ* gene fragments (δ chain) (27, 28). These random insertions and deletions directly affect the length of the mature CDR3 (28). Thus, the N-addition, V-trim, and J-trim length polymorphism distributions were also analyzed. The weighted average of the N-addition in TRG in TB patients was significantly higher than that in the controls, while the V-trim and J-trim length distributions were similar in both the TRD and TRG groups (Figures S2G,H). Finally, we analyzed the differences in the shared CDR3 sequences between the TB patients and healthy controls. The results showed that the proportion of shared CDR3 TRD sequences with the clonal size over 0.1% in TB patients was higher than these of healthy controls (Figure S2I). These findings suggest that, in TB patients, insertions and rearrangements of gene fragments in the CDR3 regions create new clones or induce the amplification of original clones.

### The BCG Vaccine Stimulates γδT Cell Activation and Modifies the Characteristics of the γδT Cell CDR3 Immune Repertoire

**Figure 3:**
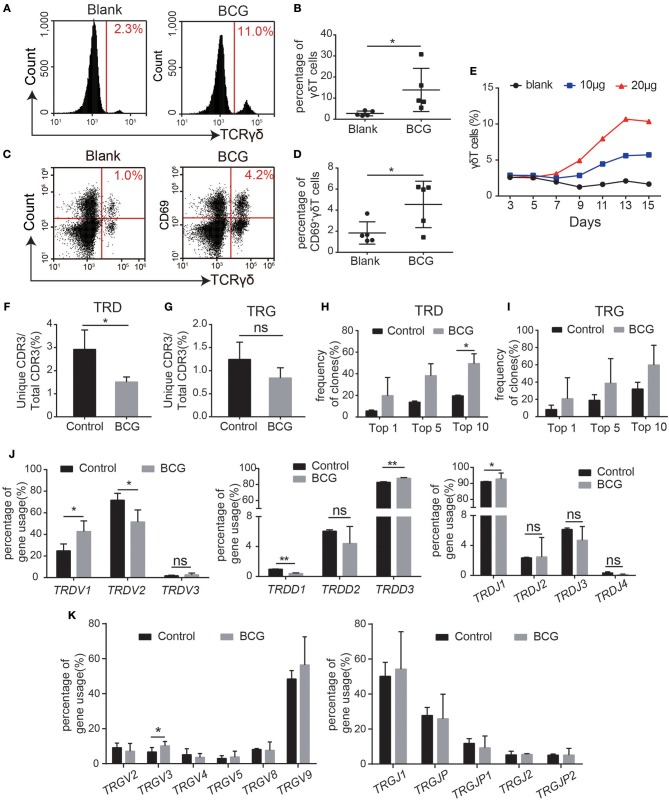
BCG treatment recapitulated the characteristics of the γδT cell immune repertoire in TB patients. **(A–E)** Flow cytometry analysis of the proportion of BCG-stimulated γδT cells and CD69^+^γδT cells **(A,C)**. Quantification of the proportion of γδT cells **(B)** and CD69^+^γδT cells **(D)**. **(E)** Average proliferation curve of γδT cells stimulated by BCG. **(F,G)** Quantification of the ratios of unique CDR3/total CDR3 of the δ **(F)** and γ **(G)** chains in the CDR3 region. **(H,I)** Frequencies of the top 1, 5, and 10 CDR3 sequences of the δ **(H)** and γ **(I)** chains in the CDR3 region. **(J)** The germline gene usage of T-cell receptor δ-chain (TRD) *V* fragments (*TRDV1, TRDV2, TRDV3*), *D* fragments (*TRDD1, TRDD2, TRDD3*), and *J* fragments (*TRDJ1, TRDJ2, TRDJ3, TRDJ4*). **(K)** The germline gene usage of T-cell receptor γ-chain (TRG) *V* fragments (*TRGV2, TRGV3, TRGV4, TRGV5, TRGV8, TRGV9*) and *J* fragments (*TRGJ1, TRGJ2, TRGJP, TRGJP1, TRGJP2*). Data represent the mean ± SD. *P < 0.05, **P < 0.01 by Student's paired *t* test.

Since it is impossible to compare the immune repertoire alteration of one individual before and after *Mtb* infection, we applied the BCG vaccine to stimulate γδT cells from healthy people *in vitro* and compared the CDR3 immune repertoire characteristics of γδT cells before and after stimulation in the same person. Thus, the effect of individual differences in the immune repertoire analysis was excluded. Flow cytometry analysis showed that the proportion of γδT cells was significantly increased after BCG stimulation (Figures 3A,B). Expression of the activation marker CD69 on γδT cells was also significantly increased after BCG treatment (Figures 3C,D). BCG treatment stimulated γδT cell proliferation in a dose-dependent manner (Figure 3E), suggesting that BCG could activate γδT cells. In TB patients, γδT cells are persistently activated and “exhausted,” resulting in decreased proportions of γδT cells in the peripheral blood from TB patients.

Next, we sought to determine whether BCG treatment modified the characteristics of the γδT cell immune repertoire. Thus, we performed high-throughput sequencing on three pairs of samples, each pair comprising γδT cells from the same individual before and after BCG treatment *in vitro* (Table 1). The results showed that TRD diversity was significantly reduced after BCG stimulation, while no significant difference was observed for TRG (Figures 3F–I). These results indicate that some specific γδT cell clones, especially TRD, were selectively expanded after BCG stimulation, consistent with the findings in TB patients, as shown in Figure 2. We also found no significant differences in the size and distribution of dominant sequences or in the distribution of CDR3 length polymorphisms (Figure S3). Next, we analyzed the frequency of the gene fragments (*VDJ*) and found an alteration in the pattern of the TRD gene fragment distribution (Figure 3J). The percentages of *hTRDV1, hTRDD3*, and *hTRDJ1* were significantly increased compared with those in the controls, while the percentages of *hTRDV2* and *hTRDD1* were significantly reduced (Figure 3J). No significant change was observed in TRG (Figure 3K). Collectively, these results, excluding interference from individual differences, are consistent with the observations in TB patients that the diversity of the γδT cell CDR3 immune repertoire was reduced and that the usage of gene CDR3 fragments was significantly altered by *Mtb* infection. Thus, we identified 10 *Mtb* infection-associated CDR3δ sequences from TB patient immune repertoires according to the screening criteria: high frequency in the TB patient immune repertoires but no or very low frequency in healthy controls (Table 2). In addition, we found several conserved amino acid sequences of the CDR3 regions, such as “ALG” (δ*1* gene) and “ACD” (δ*2* gene), at the N-terminus and “DKLI” at the C-terminus. In contrast, the inner regions of CDR3 were composed of variable sequences.

**Table 1:** General information of the peripheral blood γδT cell receptor repertoire in healthy individuals stimulated by BCG *in vitro*.

**Sample ID**	**BCG stimulation**	**Chain**	**Total reads**	**CDR3 reads**	**Unique CDR3**
1217	–	TRD	2025392	200110	7307
		TRG	2929486	332771	5395
1218	+	TRD	3168138	384309	6289
		TRG	3031580	407345	3928
1219	–	TRD	1943906	177287	5515
		TRG	2553568	281928	3487
1220	+	TRD	1969570	279616	4567
		TRG	2681846	393286	2310
1221	–	TRD	2018494	256930	5137
		TRG	2315868	295577	2587
1222	+	TRD	2214164	132647	1670
		TRG	2767270	324793	3160

*^−^No BCG stimulation. ^+^With BCG stimulation*.

**Table 2:** CDR3δ sequences in healthy controls and TB patients.

**NO**.	**Gene**	**Length**	**Amino acid sequence**	**Share information**
1	Vδ1	17aa	ALGELIRGGITYTDKLI	3 patients 0 healthy control
2	Vδ1	18aa	ALGLHKRAVLLGEFDKLI	2 patients 0 healthy control
3	Vδ2	15aa	ACDTVLGAPVADKLI	2 patients 0 healthy control
4	Vδ2	15aa	ACDTVGLGDPLDKLI	2 patients 0 healthy control
5	Vδ2	13aa	ACDPVLRVKGELI	2 patients 0 healthy control
6	Vδ2	14aa	ACDTLTGGYTDKLI	8 patients 1 healthy control
7	Vδ2	13aa	ACDTLGDTPDKLI	7 patients 3 healthy controls
8	Vδ2	14aa	ACDTLGDTPTDKLI	7 patients 2 healthy controls
9	Vδ2	14aa	ACDPLGDPYTDKLI	7 patients 0 healthy control
10	Vδ2	13aa	ACDTVGGDTDKLI	6 patients 2 healthy controls

### Identification of *Mtb*-Related Antigen Epitopes Bound to Specific CDR3δ Peptides Using the Ph.D.^TM^-12 Phage Display Peptide Library

**Figure 4:**
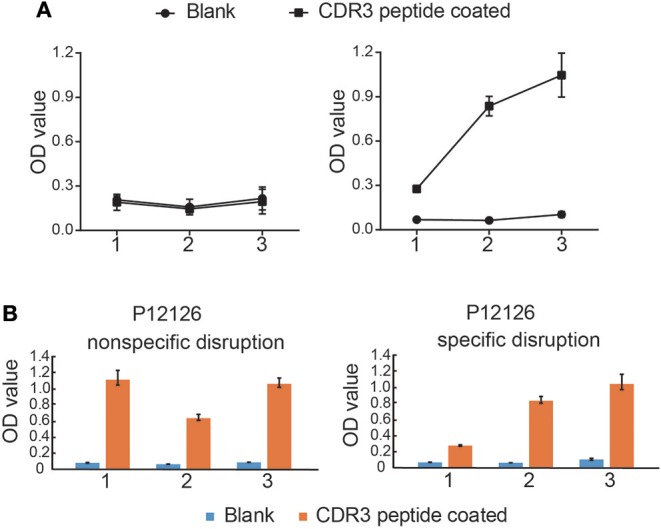
*Mtb*-related antigen epitopes bound to CDR3δ peptides screened using the Ph.D^TM^-12 Phage Display Peptide Library. **(A)** The binding curve of phage elutions to CDR3δ probes by ELISA. The left panel shows the binding of non-specifically eluted phage to the CDR3δ probes, and the right panel shows the binding of the specifically eluted phage to the CDR3δ probes. 1, 2, and 3 indicate the screening rounds. **(B)** Histograms of the binding between phage elutions to CDR3δ probes by ELISA. Data represent the mean ± SEM.

Next, we randomly selected two CDR3δ sequences from the 10 identified *Mtb* infection-associated CDR3δ sequences as probes to identify *Mtb*-related antigen epitopes using the Ph.D.™-12 Phage Display Peptide Library. We performed three rounds of enrichment, and each round utilized specific and non-specific elution methods. Phage titer analysis showed that the elution and expansion of the phage were successful (Table S3). After three rounds of screening, we detected the binding of phage clones to the probes by ELISA. The results showed that the CDR3δ probe-bound phage clones were successfully enriched by the specific elution method (Figure 4). Therefore, we used the specific elution method for all probes to screen phage clones. ELISA was performed to examine the binding between eluate and probe. Only the probe P12126 (ALGLHKRAVLLGEFDKLI) was able to significantly enrich phage clones (Figure 4 and Figure S4A). Thus, we collected the final round of phage clones that were screened and eluted with the P12126 probe. After amplification, purification and identification of these clones, the DNA size was found to be correct (195 bp) (Figure S4B). High-throughput sequencing was performed to examine the purified phage DNA, and 996 different dodecapeptide sequences were identified (Table 3). We sorted all the dodecapeptide sequences according to the sequencing frequency and obtained the top ten sequences (Table 4), in which the total frequency of the top four sequences accounted for 97.9%. BLAST alignment of these four sequences showed that sequences homologous to these dodecapeptides are present in the structural molecules of prokaryotes such as bacteria and bacilli (Table 5). This result indicates that no dodecapeptide epitope is homologous to the *Mtb* sequence. Although the probes we used were from TB patients, these probes were not specific to *Mtb*-associated proteins. In addition, these dodecapeptides are linear epitopes, while the true binding of γδT cells may be a folded conformational epitope.

**Table 3:** The reads of high-throughput sequencing after Ph.D.-12^TM^ Phage Display Peptide screening.

**Total reads**	**Average reads**	**Unique**	**Ratio (unique/total reads)**	**Length**
7099434	7127.9	996	0.01%	12aa

**Table 4:** The frequencies of the top 10 epitope peptides.

	**Count**	**Ratio (count/total reads)**	**Length**	**Amino acid sequence**
EP1	4546994	64.05%	12aa	DYHDPSLPTLRK
EP2	2044374	28.80%	12aa	HSSATKPWKLKH
EP3	250442	3.53%	12aa	TAKYLPMRPGPL
EP4	110111	1.55%	12aa	HPMHMLHKRQHG
EP5	26039	0.37%	12aa	RDYHPRDHTATW
EP6	20893	0.29%	12aa	GNNPLHVHHDKR
EP7	6614	0.09%	12aa	QVNGLGERSQQM
EP8	4531	0.06%	12aa	WHKNEANLSTRL
EP9	2513	0.04%	12aa	SPLRAVAFSGAQ
EP10	2405	0.03%	12aa	AYHDPSLPTLRK

**Table 5:** Matched proteins with the identified epitope peptides.

**No**.	**Sequence**	**Reference**	**Matched protein [species]**	Matching manner^*^
EP1	DYHDPSLPTLRK	CBQ73568.1	Related to molybdenum cofactor sulfurase [Sporisorium reilianum SRZ2]	YHDPSL••LRK
		WP_034895250.1	Rhodanese-like domain-containing protein [Erwinia typographi]	HDPSLPTLR
		WP_018396733.1	WYL domain-containing protein [filamentous cyanobacterium ESFC-1]	DY–PSLPTLRK
EP2	HSSATKPWKLKH	XP_012068215.1	PREDICTED: A-kinase anchor protein 17A [Jatropha curcas]	SSATKP—WKLKH
		XP_001010658.2	STOP protein [Tetrahymena thermophila SB210]	ATKPWKLK
		XP_002302406.2	Hypothetical protein POPTR_0002s11910g, partial [Populus trichocarpa]	SSATKP—WKLK
EP3	TAKYLPMRPGPL	OJV00680.1	Hypothetical protein BGO12_13510 [Verrucomicrobia bacterium 61-8]	KYLPMR••PL
		WP_013075631.1	Fumarylacetoacetate (FAA) hydrolase [Kyrpidia tusciae]	TA•YLP+RPG
		XP_006405121.1	Hypothetical protein EUTSA_v10000546mg [Eutrema salsugineum]	T•KY•PMRPG
EP4	HPMHMLHKRQHG	WP_042297702.1	PadR family transcriptional regulator [Paraburkholderia bannensis]	HPMHMLH
		XP_013762827.1	Nuclear liminteractor-interacting factor [Thecamonas trahens ATCC 50062]	HP–M+MLH••QHG
		XP_002507500.1	Sister chromatid arm cohesin [Micromonas commoda]	HPMH+L•KRQ

*The BLAST results of matched proteins with five identified epitope peptides*.

* *Matching shows the motif-matched format based on the primary sequence of proteins*.

*^•^A different amino acid in the position*.

*^+^A similar amino acid in the position*.

*^−^A redundant or an absent amino acid in the position*.

### Identification of *Mtb* Antigen-Related Protein Ligands Bound to Specific CDR3δ Peptides Using an *Mtb* Proteome Chip

**Figure 5:**
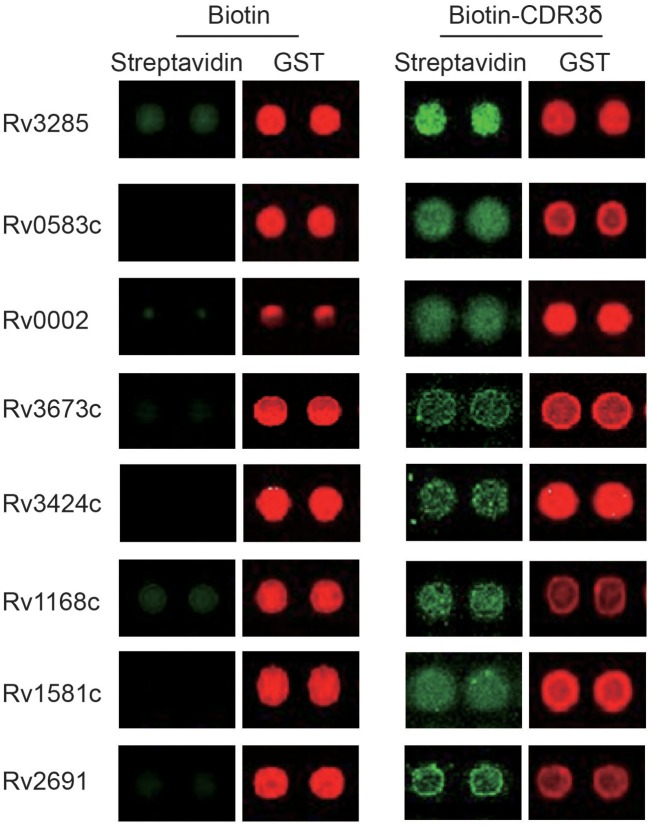
*Mtb* antigen-related protein ligands bound to CDR3δ peptides screened using the *Mtb* proteome chip. Red fluorescence marks the GST tag, indicating the presence of the corresponding protein on the chip. Green fluorescence marks the binding of the probe. Lines 1, 3, 5, and 7 act as the bio-CDR3 peptide, lines 2, 4, 6, and 8 act as the biotin control.

To identify CDR3δ peptide-bound *Mtb* antigen-related protein ligands, a biotin tag was added to the N-terminus of the selected CDR3δ peptides to served as a probe to screen protein ligands using an *Mtb* proteome chip, which covers 4,262 TB proteins (29). Eight TB proteins were identified with high binding activities to the CDR3δ peptide probes (Figure 5). As shown in Table 6, three proteins were identified by the probe P12126. There are very few studies on these three proteins. Only Rv0002 protein was reported to be a DNA polymerase III subunit β (30). Thus, we chose Rv0002 for further experiments.

**Table 6:** Identified potential ligands of γδT cells by screening the *Mycobacterium tuberculosis* protein chip.

**Sequence of probes**	**Protein**	**Protein function**
ALGELIRGGITYTDKLI	Rv3673c	Oxidoreductase activity; Antioxidant activity; Protein disulfide oxidoreductase activity; Cell redox homeostasis; Cellular response to oxidative stress; Oxidation-reduction process; Protein folding; Sulfate assimilation.
	Rv2691	Potassium ion transmembrane transporter activity
	Rv3424c	Unknown
	Rv3285	Acetyl-CoA carboxylase activity; Biotin carboxylase activity; Metal ion binding; Ropionyl-CoA carboxylase activity; Carbon fixation; Growth.
	Rv1168c	Unknown
ALGLHKRAVLLGEFDKLI	Rv0002	3′-5′ exonuclease activity; DNA-directed DNA polymerase activity; Nucleotidyl transferase, Transferase
	Rv0583c	Unknown
	Rv1581c	Unknown

### Rv0002 Could Stimulate γδT Cell Activation and Proliferation *in vitro*

**Figure 6:**
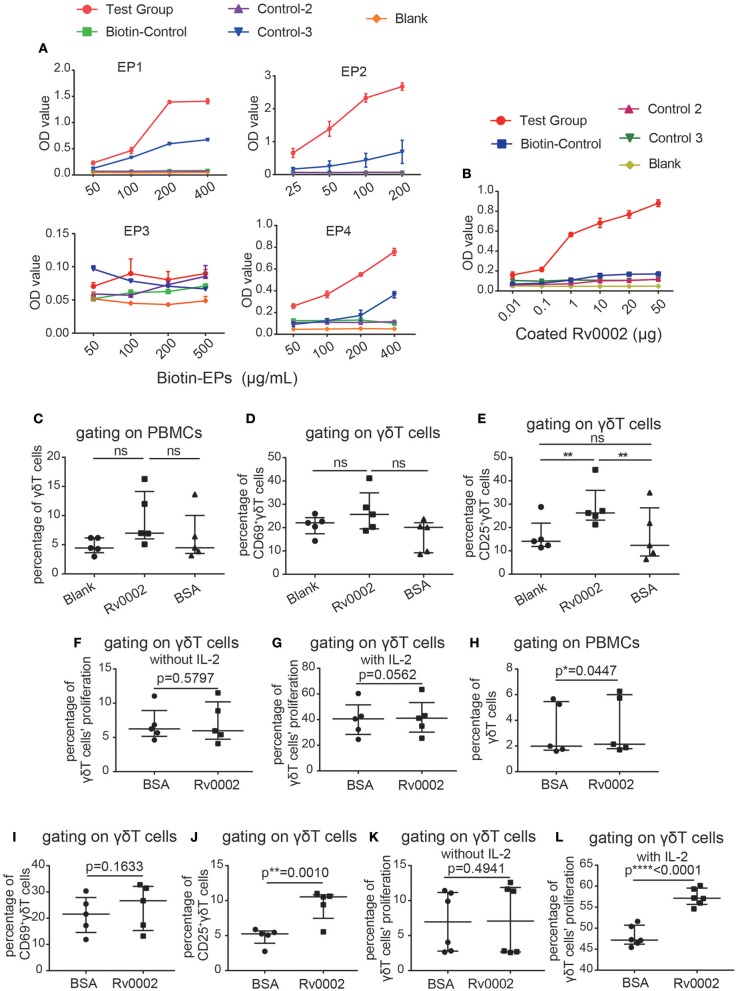
Epitopes/Rv0002 stimulated the activation and proliferation of γδT cells *in vitro*. **(A,B)** Binding curve of the epitopes **(A)** and the Rv0002 protein to the CDR3δ probe **(B)** by ELISA. **(C–E)** Quantification of the proportions of γδT cells **(C)**, CD69^+^γδT cells **(D)** and CD25^+^γδT cells **(E)** in PBMCs of healthy people stimulated with Rv0002 (20 μg/mL). Blank: PBMCs cultured with IL-2 alone as a negative control; BSA: immobilized BSA (20 μg/mL) as a randomized control; Rv0002: immobilized Rv0002 protein. **(F,G)** Quantification of the proliferation of γδT cells in the PBMCs of healthy people stimulated with Rv0002 (20 μg/mL) using CFSE staining. The proportion of γδT cells was more than 85%. The final concentration of IL-2 was 40 IU/mL. **(H–J)** Quantification of the proportions of γδT cells **(H)**, CD69^+^γδT cells **(I)**, and CD25^+^γδT cells **(J)** in the PBMCs of TB patients stimulated with Rv0002 (20 μg/mL). BSA: immobilized BSA (20 μg/mL) as a randomized control; Rv0002: immobilized Rv0002 protein. **(K,L)** Quantification of the proliferation of γδT cells in the PBMCs of TB patients stimulated with Rv0002 (20 μg/mL) using CFSE staining. The proportion of γδT cells was more than 85%. The final concentration of IL-2 was 40 IU/mL. Data represent the median with interquartile range. *P < 0.05, **P < 0.01 by Student's paired *t* test.

Next, to determine whether the identified *Mtb* antigen-related proteins were genuine γδT cell ligands capable of stimulating γδT cell activation and proliferation, we performed functional verification of the top four epitopes and the Rv0002 protein *in vitro*. Four epitopes (EP1, EP2, EP3, EP4) were synthesized and assayed for binding to the probe P12126 by ELISA. Three peptides (EP1, EP2, EP4) bound well with the probe (Figure 6A). At the same time, we expressed the Rv0002 protein in *E. coli* (Figure S5) and verified its binding to the probe by ELISA (Figure 6B). Next, we examined whether Rv0002 proteins could functionally stimulate γδT cell activation and proliferation *in vitro*. Immobilized Rv0002 significantly promoted expression of the activation marker CD25, indicating that Rv0002 protein could functionally stimulate γδT cell activation (Figures 6C–E and Figures S6A–C). Next, we examined the effect of Rv0002 on γδT cell proliferation via CFSE staining. The results showed that, although the percentage of γδT cells in the Rv0002-treated group was slightly higher than that in the control group, the difference was not statistically significant (Figures 6F,G and Figures S6D–E). These results suggest that the Rv0002 protein is weakly antigenic toward γδT cells *in vitro*.

We also used the Rv0002 protein to stimulate γδT cells of TB patients. The results showed that the Rv0002 protein was able to amplify γδT cells from the PBMCs of TB patients *in vitro* (Figure 6H and Figure S7A). Expression of the activation index CD25 was also significantly higher in the TB patient group than in the control group (Figures 6I,J and Figures S7B,C). Under conditions of 40 IU/mL of IL-2 in the culture medium, Rv0002 significantly promoted γδT cell proliferation (Figures 6K,L and Figures S7D,E). Taken together, these results demonstrate that Rv0002 can serve as an *Mtb* antigen to stimulate γδT cell activation and proliferation.

## Discussion

γδT cells account for only a small percentage (1~5%) of lymphocytes in human peripheral blood but are abundant in epithelial tissues. γδT cells can initiate a rapid immune response during early infection and have MHC non-restriction. The rearrangement of γδT cells through the *V-(D)-J* junction region (the CDR3 region) forms a highly diverse TCRγδ repertoire. In our previous work, high-throughput sequencing was used to analyze the characteristics of the TCRγδ CDR3 repertoire in human peripheral blood, confirming changes in the γδT cell repertoire under different conditions (14, 27). Therefore, in the present study, we analyzed the characteristics of the TCRγδ CDR3 repertoire in the peripheral blood of TB patients using high-throughput sequencing. We found that the diversity of the TCRγδ CDR3 repertoire was reduced in TB patients, suggesting that γδT cell responses to TB were selectively amplified among the original clones. The frequency of the gene fragments changed mainly in the *V* segment. To eliminate the interference caused by individual differences in the repertoire analysis, we used BCG to stimulate the peripheral blood lymphocytes of healthy people *in vitro* and collected cells with and without BCG stimulation from the same individuals as paired samples to perform high-throughput sequencing analysis. The results showed that the changes in the immune repertoire after stimulation with BCG were consistent with those of TB patients, and the changes in TRD were the most obvious. This result further proves that CDR3δ plays a key role in antigen recognition. Therefore, we screened out 10 specific CDR3δ dominant sequences based on the results of the repertoire of TCRγδ CDR3 regions in TB patients.

BCG does not fully mimic *Mtb*, but we were unable to obtain peripheral blood cell samples from patients before they became infected with *Mtb*, and the sources and use of intact *Mtb* are limited. Therefore, accurately reflecting changes in the human γδT cell repertoire before and after *Mtb* infection remains a limitation.

The screening method used in this study was based on specific CDR3δ probes. This method was previously used to successfully screen the tumor-associated ligand human mutS homolog 2 (hMSH2) (31) and the BCG protein oxidative stress response regulatory protein (OXYS) (32), which are recognized by γδT cells. In this study, we used the *Mtb*-specific CDR3δ dominant sequence as probes to screen phage clones using Ph.D.™-12 Phage Display Peptide Library. Three of the dodecapeptide epitopes that specifically bound to the probe were identified by high-throughput sequencing. Unfortunately, we found no domains of *Mtb* protein molecules that matched the three epitope peptides through BLAST homology alignments, and the three epitope peptides did not significantly activate γδT cells in the PBMCs of healthy people. This result may be due to the linear structure of epitope peptides, in contrast to the conformational epitopes recognized by γδT cells (33). In addition, whether the modified epitope peptides are recognized by γδT cells requires further verification.

Through the *Mtb* proteome chip, we identified eight *Mtb* proteins that specifically bound to probes. Because the P12126 probe could able to bind both *Mtb* protein and phage clones, we selected the Rv0002 protein, which bound P12126. *In vitro* functional experiments showed that Rv0002 activated γδT cells in the peripheral blood of healthy people and TB patients and stimulated γδT cell proliferation in the peripheral blood of TB patients (Figure 6). Because the P12126 probe belongs to the *V*δ*1* gene sequence, we speculate that Rv0002 may have a more pronounced effect on Vδ1γδT cells. However, the Rv0002 protein is expressed in the bacterial nucleus. How do γδT cells recognize it? We believe that there may be two ways. First, some *Mtb* proteins might be ectopically expressed to the bacteria surface under stress or drug treatment. The second possibility is that Rv0002 proteins were released from dead *Mtb*, which were killed by drugs or immune cells (such as macrophage or NK cells) in the body. However, further experiments are essential to elucidate the biological action between Rv0002 and Vδ1γδT cells.

In conclusion, in this study, we used high-throughput sequencing technology to analyze the characteristics of the TCRγδ CDR3 repertoire in TB patients and found that the diversity of CDR3 regions was significantly reduced, especially in the TRD chain. The adoption of gene fragments was significantly altered, especially for *V* fragments. The *Mtb* protein Rv0002 recognized by γδT cells was identified from an *Mtb* proteome chip using an *Mtb*-specific CDR3δ probe. This study provides a molecular basis for the mechanism of γδT cell-mediated resistance against *Mtb* infection.

## Data Availability Statement

All datasets generated for this study are included in the manuscript/Supplementary Files.

## Ethics Statement

This study was carried out in accordance with the recommendations of the Ethical Committee of the Chinese Academy of Medical Sciences with written informed consent from all subjects. All subjects gave written informed consent in accordance with the Declaration of Helsinki. The protocol was approved by the Ethical Committee of the Chinese Academy of Medical Sciences.

## Author Contributions

YL, XW, JW, DT, HC, and MW performed the experiments. YL and XW analyzed the data and performed the statistics. YL, JZ, and WH designed the experiments and edited the manuscript.

### Conflict of Interest

The authors declare that the research was conducted in the absence of any commercial or financial relationships that could be construed as a potential conflict of interest.
