# Supplementary material for: Identification of the Ligands of TCRγδ by Screening the Immune Repertoire of γδT Cells From Patients With Tuberculosis
Source: Front Immunol. 2019 Sep 24;10:2282. doi: 10.3389/fimmu.2019.02282 (PMC6769167; doi:10.3389/fimmu.2019.02282)
Supplement: Supplementary file 1 [file Data_Sheet_1.pdf]

Supplementary Material

Identification of the ligands of TCRγδ by screening the Immune Repertoire of γδT Cells from Patients with Tuberculosis

Yuxia Li[1], Xinfeng Wang[2], Da Teng[1], Hui Chen[1], Maoshui Wang[2], Junling Wang[2], Jianmin Zhang[1,*], Wei He[1,*]

[1]Department of Immunology, Research Center on Pediatric Development and Diseases, Institute of Basic Medical Sciences, Chinese Academy of Medical Sciences and School of Basic Medicine, Peking Union Medical College, State Key Laboratory of Medical Molecular Biology, Beijing, China

[2]Department of Laboratory Medicine, Shandong Provincial Chest Hospital, Jinan, China.

***Correspondence should be addressed to:**

Jianmin Zhang, Ph.D. or Wei He, M.D.

Department of Immunology, Research Center on Pediatric Development and Diseases, Institute of Basic Medical Sciences, Chinese Academy of Medical Sciences and School of Basic Medicine, Peking Union Medical College, State Key Laboratory of Medical Molecular Biology, Beijing, 100005, China

Tel.: +86-10-69156474

E-mail: jzhang42@163.com or heweingd@126.com

Supplementary Figures

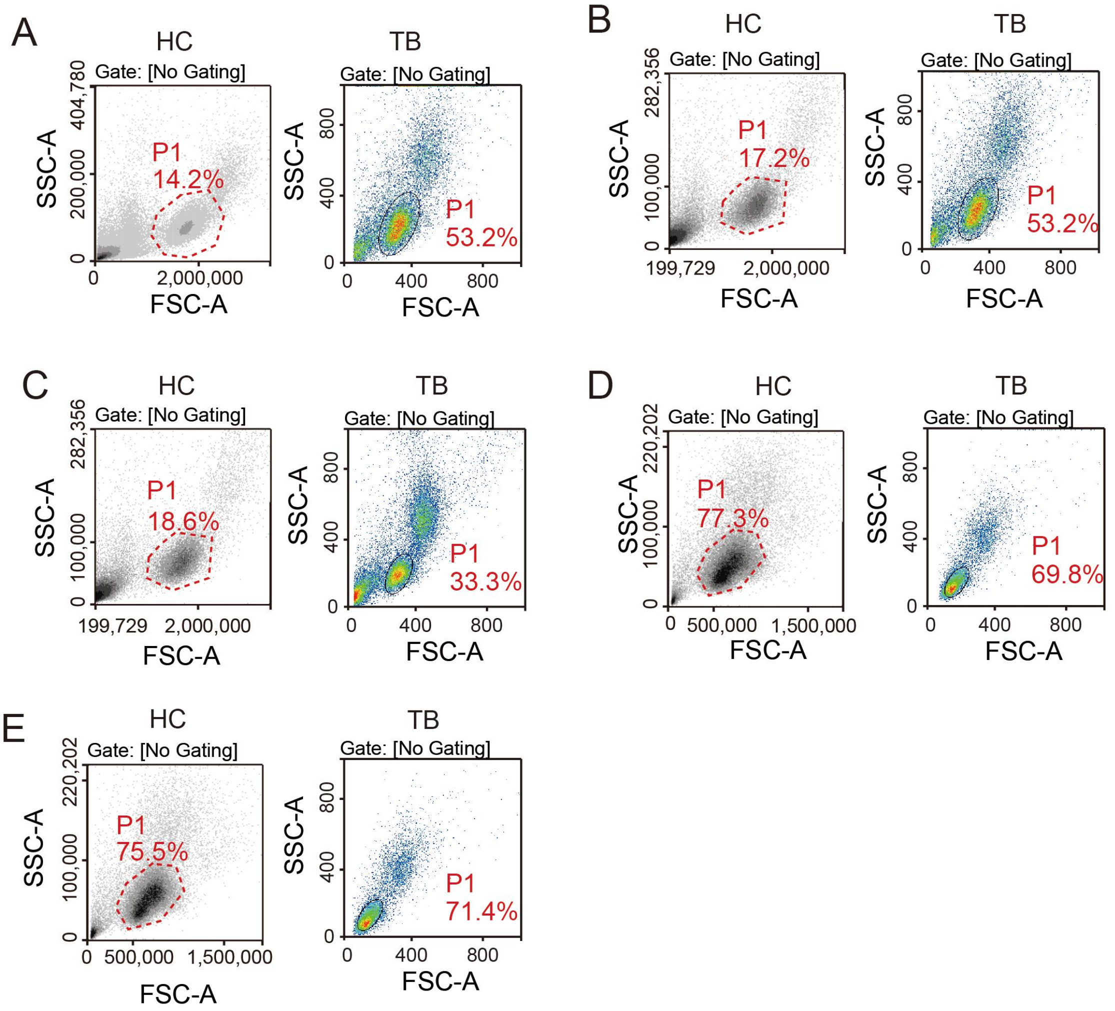

Figure S1 The gating strategy for flow cytometry analysis in Figure 1. As shown in the figures, we set the gates for flow cytometry analysis of γδT cells (A), Vδ2γδT cells (B), $CD27^+$γδT cells (C), of IFN-γ^+ γδT cells (D) and IL-$17A^+$γδT cells (E) in PBMCs from healthy controls (HC) and TB patients (TB). The P1 represent the proportions of PBMCs.

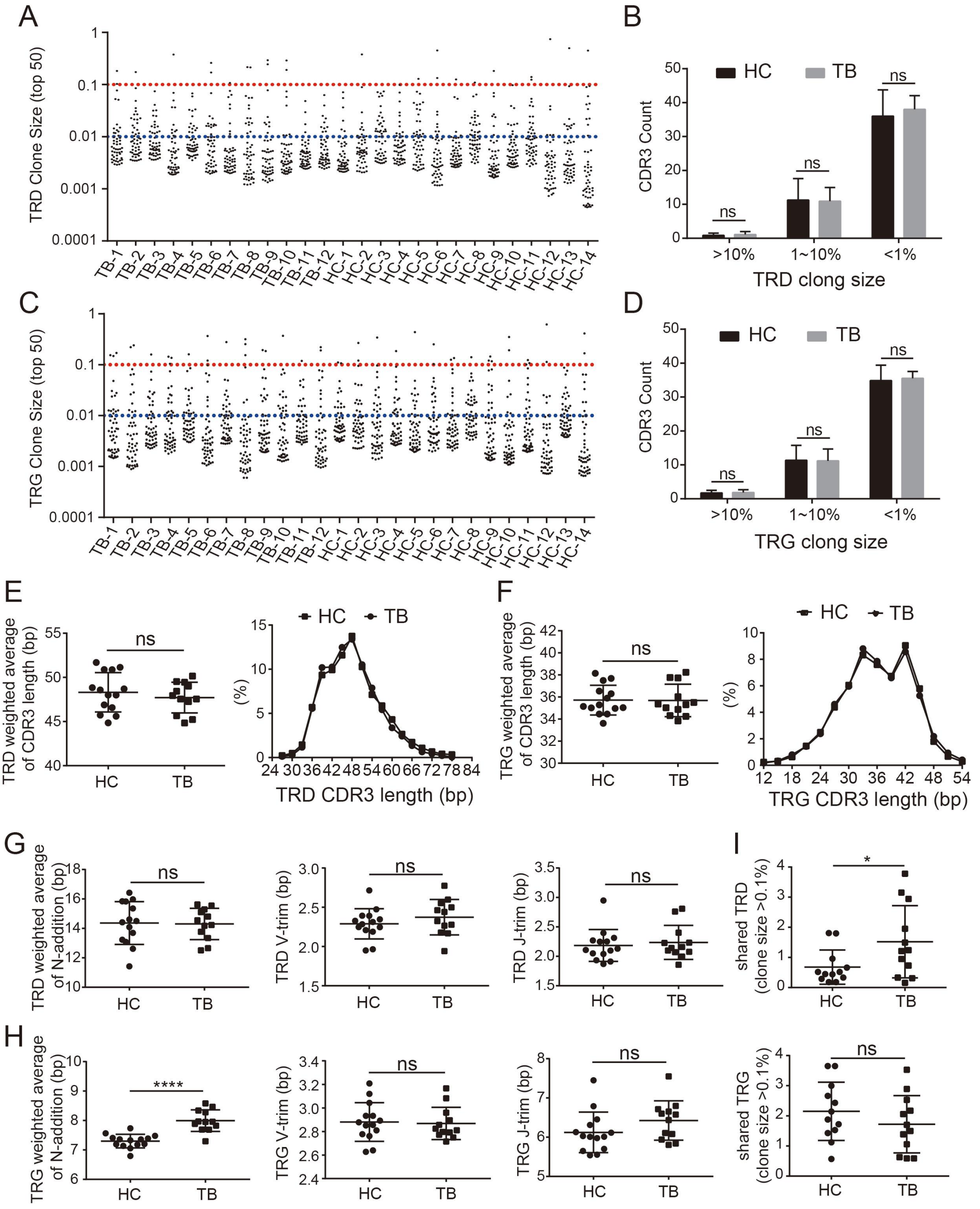

Figure S2 Clone size, CDR3 length, N-addition, V-trim, and J-trim length distributions were similar in TB patients and healthy individuals. (**A** and **C**). Scatter diagram of the distribution of the top 50 CDR3 clones of the δ (**A**) and γ (**C**) chains in healthy controls and TB patients. Clone size: Frequency of each CDR3 sequence/total CDR3 reads. (**B** and **D**). Quantification of the percentage of the top 50 CDR3 sequences of the δ (**B**) and γ (**D**) chains by

clone size (>10%, 1~10%, <1%). (**E** and **F**). Distribution charts of T-cell-receptor δ-chain (TRD) CDR3 lengths (**E**) and T-cell-receptor γ-chain (TRG) CDR3 lengths (**F**) in healthy controls and TB patients. Left: the weighted average of the CDR3 length distribution. Right: the average length distribution curve of healthy controls and TB patients. (**G** and **H**). Distributions of TRD (**G**) and TRG (**H**), including N-addition, V-trim, and J-trim lengths in the CDR3 region of γδT cells. Left: weighted average of N-addition lengths of each distribution. Middle: analysis of V-trim in healthy controls and TB patients. Right: analysis of J-trim in healthy controls and TB patients. (**I**). Proportions of shared CDR3 TRD and TRG sequences with the clonal size > 0.1% from TB patients and healthy controls. Data represent the mean ± SD. *, P<0.05; ***, P<0.001 by Student's *t* test.

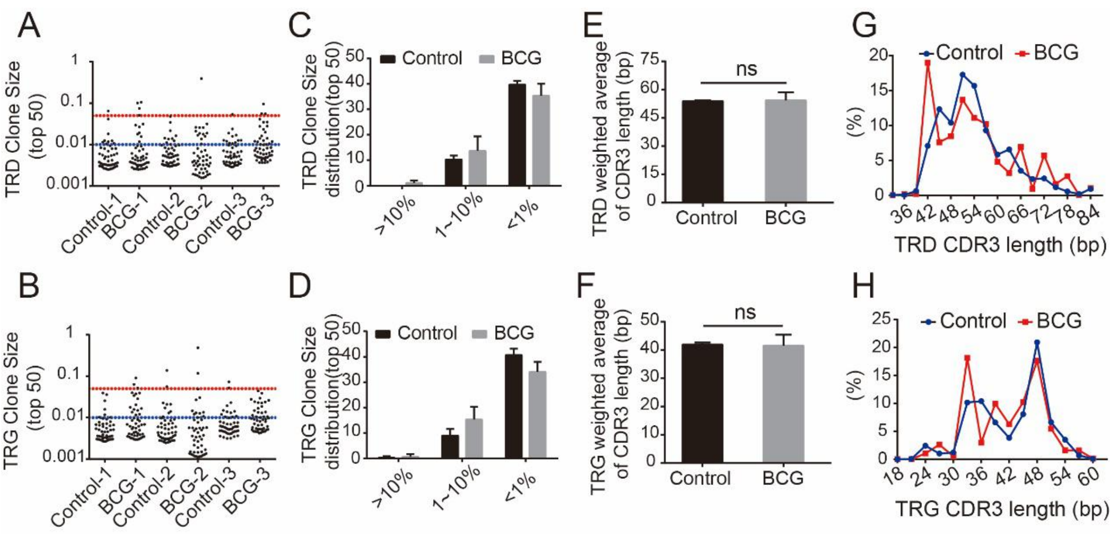

Figure S3 Clone size and length polymorphism distributions of the γδT cell CDR3 region were similar in healthy individuals with/without BCG stimulation. (**A** and **B**). Scatter diagram of the distribution of the top 50 CDR3 clones of the δ (**A**) and γ (**B**) chains with or without BCG stimulation. (**C** and **D**) Quantification of the percentage of the top 50 CDR3 sequences of the δ (**C**) and γ (**D**) chains by clone size (>10%, 1~10%, <1%). (**E** and **F**). The weighted average distribution of TRD (**E**) and TRG (**F**) CDR3 lengths with or without BCG stimulation. (**G** and **H**). The average length distribution curve of TRD (**G**) and TRG (**H**) CDR3 lengths. Data represent the mean±SD. $^*P<0.05$ by Student’s *t* test.

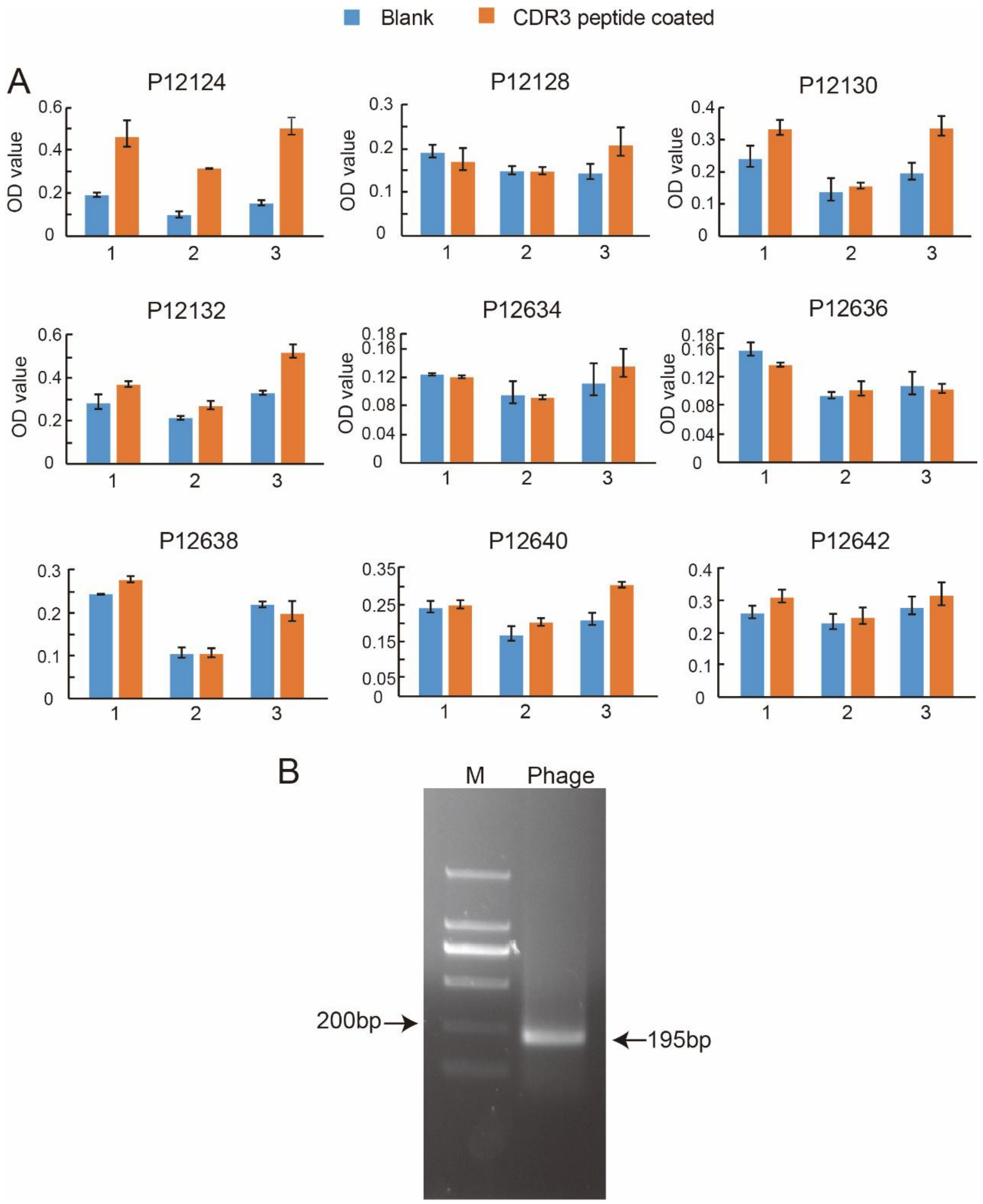

Figure S4 CDR3δ probes excluding P12126 could not enrich phage clones. (**A**). Binding activities of phage elution to nine different CDR3δ probes by ELISA. The images show the specific eluted phage to the CDR3δ probes. 1, 2, 3 represent the screening round. (**B**). The purified products of phage were detected by agarose gel electrophoresis. M: DNA Marker, Phage: The purified phage products. The data are presented as the mean ± SEM.

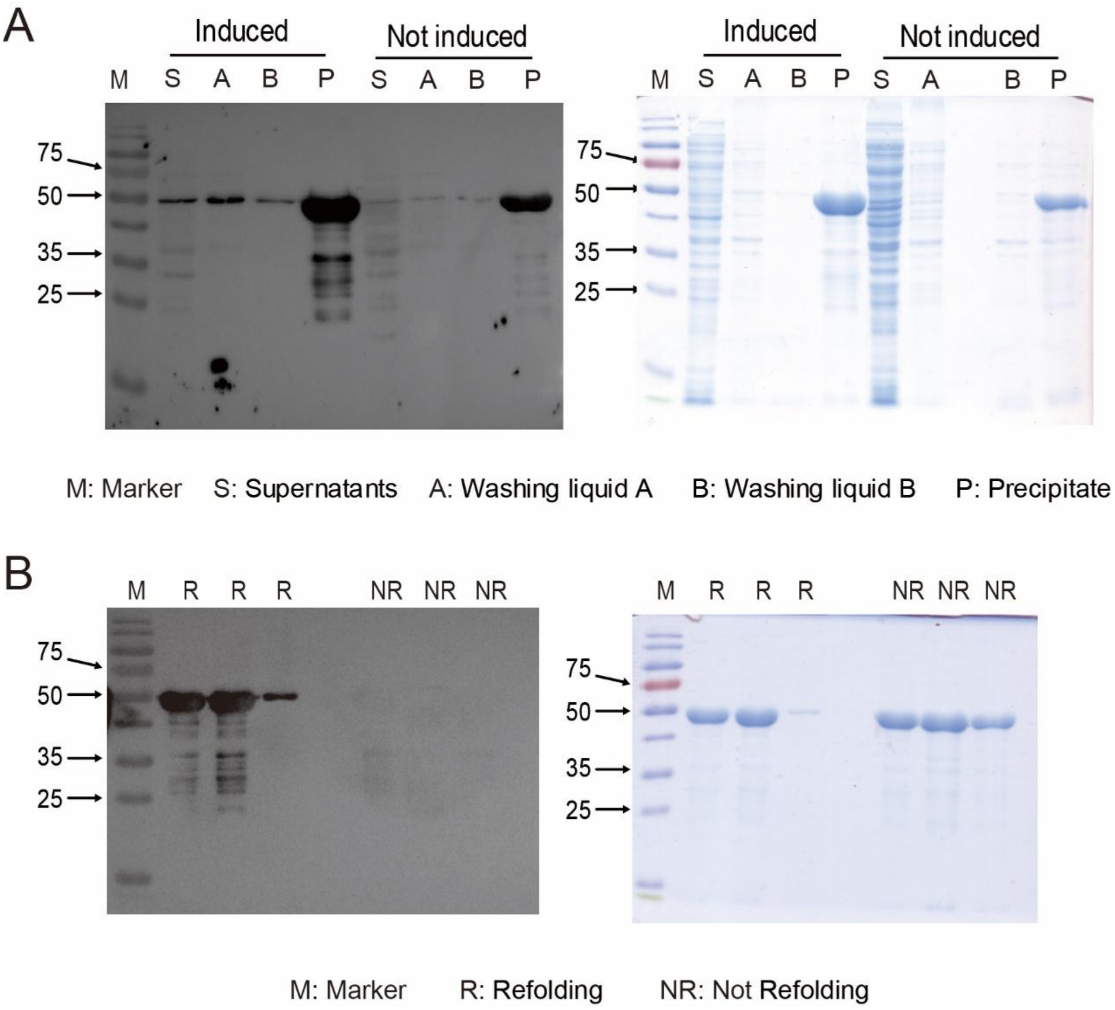

Figure S5 Rv0002 protein detected using SDS-PAGE and Western blotting. (**A**). Identification of Rv0002 protein before renaturation by Coomassie blue staining and Western blotting. (**B**). Identification of Rv0002 protein after renaturation by Coomassie blue staining and Western blotting. Left: Western blot using anti-His tag mouse mAb as the primary antibody. Right: Coomassie blue staining.

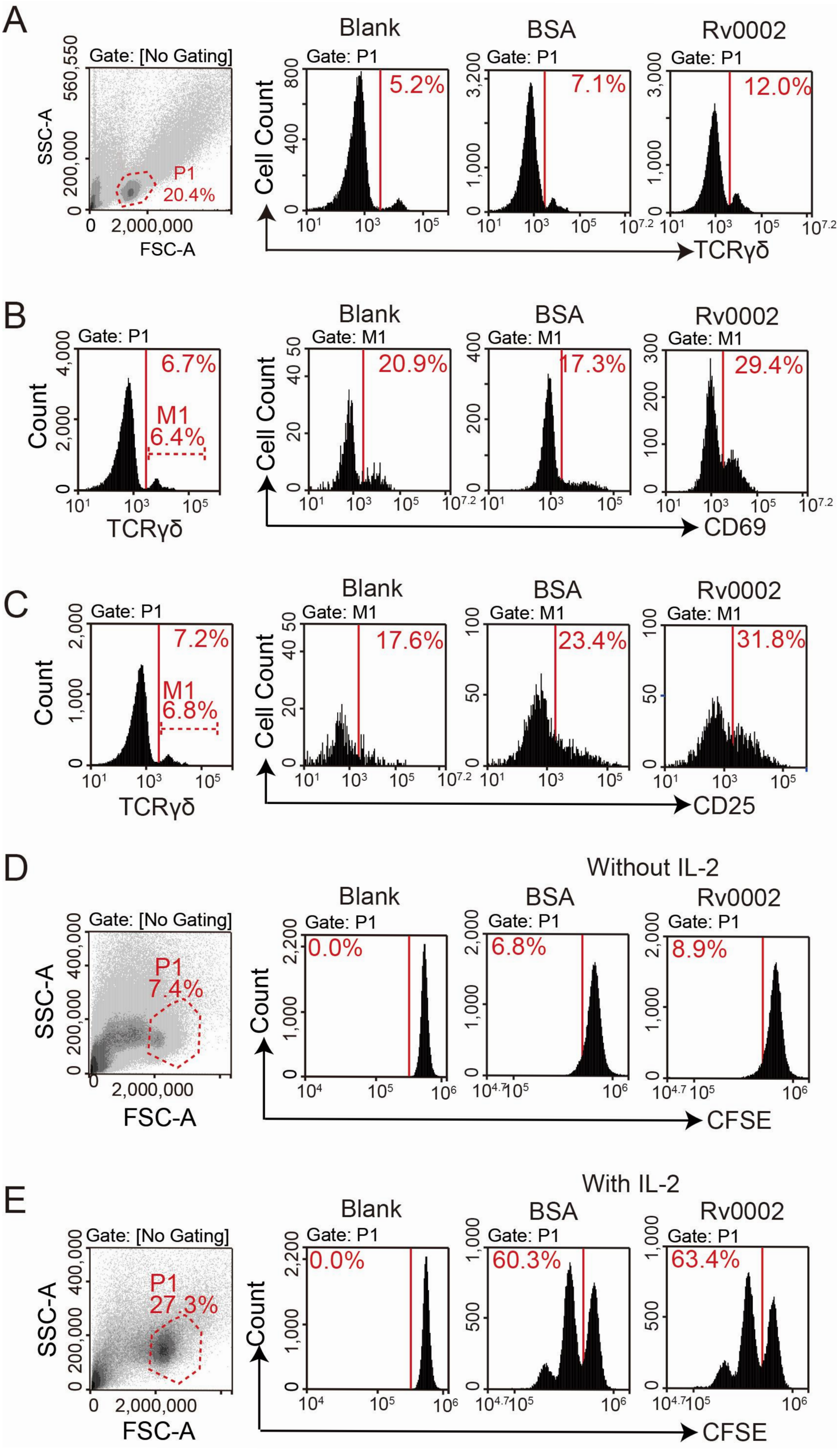
A
Gate: [No Gating]
SSC-A
FSC-A
P1
20.4%
Blank
BSA
Rv0002
Gate: P1
5.2%
7.1%
12.0%
Cell Count
TCRγδ
B
Gate: P1
Count
6.7%
M1
6.4%
Gate: M1
20.9%
17.3%
29.4%
CD69
C
7.2%
6.8%
17.6%
23.4%
31.8%
CD25
D
Without IL-2
P1
7.4%
0.0%
6.8%
8.9%
CFSE
E
With IL-2
P1
27.3%
0.0%
60.3%
63.4%

Figure S6 Activation and proliferation of γδT cells in the PBMCs of healthy people detected by flow cytometry. (**A**-**C**). Flow cytometry analysis of the proportions of γδT cells, $CD69^{+}$γδT cells and $CD25^{+}$γδT cells in the PBMCs of healthy people stimulated with Rv0002 (20 μg/mL). P1: The proportions of PBMCs; M1: The proportions of total γδT cells in P1; Blank: PBMCs cultured with IL-2 alone as a negative control; BSA: immobilized BSA (20 μg/mL) as a randomized control; Rv0002: immobilized Rv0002 protein. (**D** and E). Flow cytometry analysis of the proliferation of γδT cells in the PBMCs of healthy people stimulated with Rv0002 (20 μg/mL) using CFSE staining. The proportion of γδT cells was more than 85%. P1: The proportions of PBMCs. BSA: immobilized BSA (20 μg/mL) as a randomized control; Rv0002: immobilized Rv0002 protein. (**D**). No IL-2 was added. (**E**). The final concentration of IL-2 was 40 IU/mL.

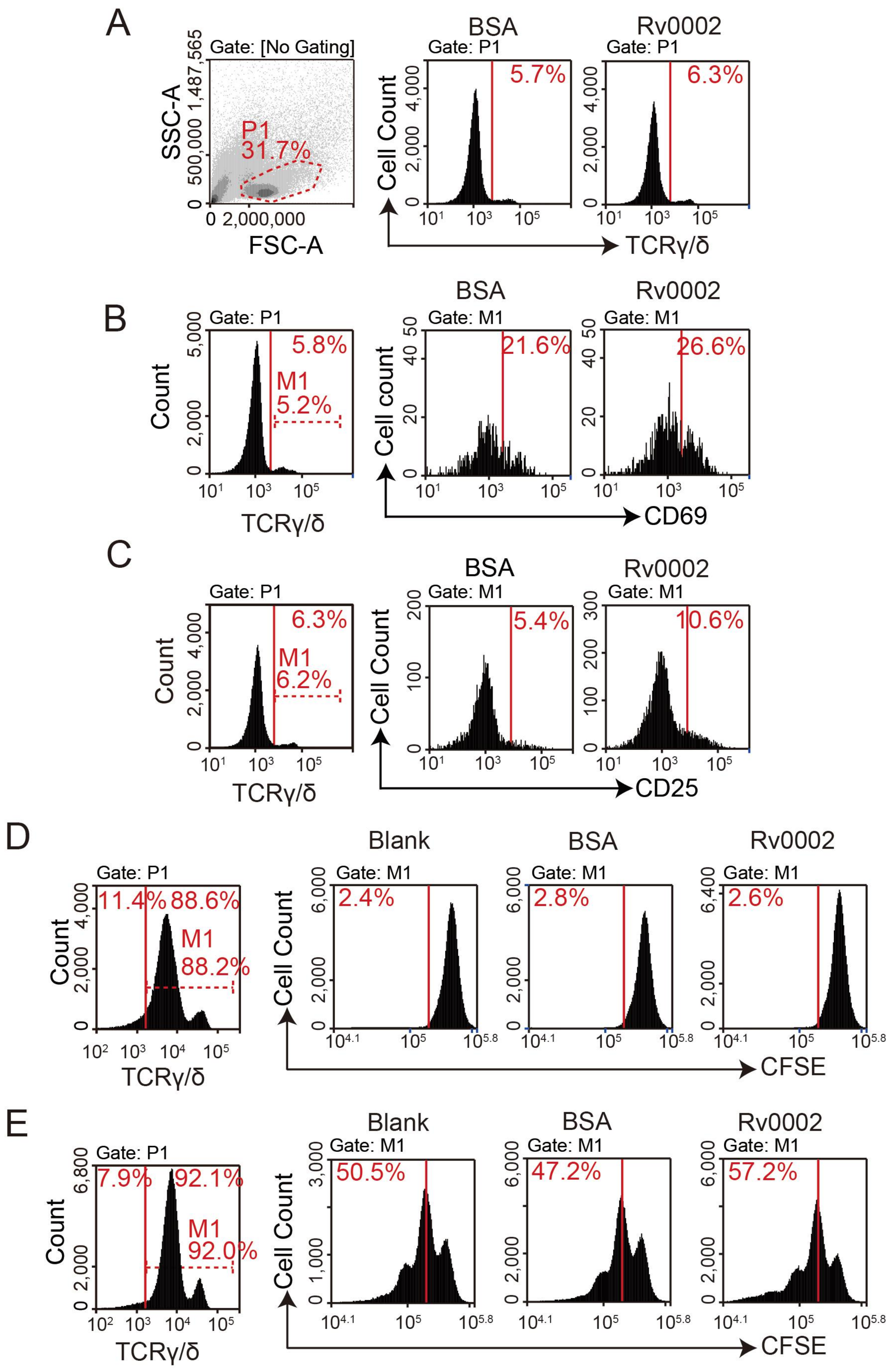

Figure S7 Activation and proliferation of γδT cells in the PBMCs of TB

patients detected by flow cytometry. (**A**-**C**). Flow cytometry analysis of the proportions of γδT cells, CD69$^+$γδT cells and CD25$^+$γδT cells in the PBMCs of TB patients stimulated with Rv0002 (20 μg/mL). P1: The proportions of PBMCs; M1: The proportions of total γδT cells in P1; Blank: PBMCs cultured with IL-2 alone as a negative control; BSA: immobilized BSA (20 μg/mL) as a randomized control; Rv0002: immobilized Rv0002 protein. (**D** and **E**). Flow cytometry analysis of the proliferation of γδT cells in the PBMCs of TB patients stimulated with Rv0002 (20 μg/mL) using CFSE staining. The proportion of γδT cells was more than 85%. M1: The proportions of CFSE-labeled γδT cells; Blank: negative control; BSA: immobilized BSA (20 μg/mL) as a randomized control; Rv0002: immobilized Rv0002 protein. (**D**). No IL-2 was added. (**E**). The final concentration of IL-2 was 40 IU/mL.

Supplementary Tables

Table S1: General information of healthy controls and TB patients

Health controls		TB patients			
No.	Age	No.	Age	Mycobacterium tuberculosis culture	sputum acid-fast stain
1	36-40	1	26-30	Positive	Negative
2	31-35	2	21-25	Positive	1+
3	26-30	3	21-25	Positive	2+
4	51-55	4	51-55	Positive	4+
5	61-65	5	36-40	Positive	4+
6	46-50	6	26-30	Positive	3+
7	51-55	7	16-20	Positive	-
8	31-35	8	16-20	Positive	Negative
9	26-30	9	61-65	Positive	Negative
10	51-55	10	51-55	Positive	Negative
11	41-45	11	66-70	Positive	3+
12	41-45	12	66-70	Positive	Negative
13	51-55	13	51-55	Positive	Negative
14	41-45	14	16-20	Positive	Negative
15	56-60				

Table S2: General information of γδT cell receptor repertoire from health controls and TB patients

Simple	Chain	Reads	CDR3	Unique CDR3	Simple	Chain	Reads	CDR3	Unique CDR3
Con-1	TRD	373326	361236	4075	Con-1	TRG	377431	259806	1563
Con-2	TRD	445120	419981	2435	Con-2	TRG	506040	372858	2294
Con-3	TRD	478051	469084	4946	Con-3	TRG	499263	274376	2760
Con-4	TRD	535236	523434	3894	Con-4	TRG	497698	197744	355
Con-5	TRD	511072	495639	11887	Con-5	TRG	474982	343205	4904
Con-6	TRD	511354	481449	2097	Con-6	TRG	523836	357904	1996
Con-7	TRD	554121	541397	3862	Con-7	TRG	889662	562292	2392
Con-8	TRD	803127	781324	5772	Con-8	TRG	2806163	2157882	1998
Con-9	TRD	339629	303365	6102	Con-9	TRG	723175	514096	4758
Con-10	TRD	715201	705559	2064	Con-10	TRG	1255041	736367	1879
Con-11	TRD	183535	180418	1108	Con-11	TRG	1961764	1412655	2102
Con-12	TRD	1445952	1436413	3119	Con-12	TRG	1446048	789581	2495
Con-13	TRD	11323265	11246502	4147	Con-13	TRG	3607817	2040305	1864
Con-14	TRD	628012	617837	14247	Con-14	TRG	612696	457491	5609
TB-1	TRD	629717	622928	2179	TB-1	TRG	4234700	3930065	3884
TB-2	TRD	1407176	1389011	2514	TB-2	TRG	4411041	3751990	2608
TB-3	TRD	3269905	3252458	9000	TB-3	TRG	3253971	2751630	6608
TB-4	TRD	1440396	1431412	1482	TB-4	TRG	2528273	2061711	1244
TB-5	TRD	418163	405323	3327	TB-5	TRG	3413204	2853047	4055
TB-6	TRD	1306426	1275146	8852	TB-6	TRG	2906213	2407102	5442
TB-7	TRD	1828615	1792983	6988	TB-7	TRG	3521262	3144765	3756
TB-8	TRD	2793954	2781636	2732	TB-8	TRG	3042965	2459210	1445
TB-9	TRD	2686663	2644615	12590	TB-9	TRG	2733315	1677233	6026
TB-10	TRD	2722359	2701401	3844	TB-10	TRG	6668883	5869229	3043
TB-11	TRD	1929630	1902195	21144	TB-11	TRG	2249420	1869780	10874
TB-12	TRD	612773	608512	2522	TB-12	TRG	2270095	1759583	3215

Table S3: Phage titering in each round of panning

Round	No amplify	Amplified	Round	No amplify	Amplified
Probe	ALGLHKRAVLLGEFDKLI				
	Specific disruption			Nonspecific disruption	
1	8.60×10^{6}pfu/ml	8.70×10^{12}pfu/ml	1	2.89×10^{7}pfu/ml	8.00×10^{12}pfu/ml
2	1.65×10^{6}pfu/ml	1.77×10^{12}pfu/ml	2	1.00×10^{7}pfu/ml	4.6×10^{12}pfu/ml
3	1.46×10^{6}pfu/ml	1.23×10^{12}pfu/ml	3	8.63×10^{7}pfu/ml	2.29×10^{12}pfu/ml
Probe	ACDTVLGAPVADKLI				
	Specific disruption			Nonspecific disruption	
1	2.10×10^{7}pfu/ml	7.00×10^{12}pfu/ml	1	2.48×10^{7}pfu/ml	6.30×10^{12}pfu/ml
2	2.00×10^{6}pfu/ml	3.47×10^{12}pfu/ml	2	2.05×10^{6}pfu/ml	7.00×10^{12}pfu/ml
3	3.60×10^{7}pfu/ml	2.06×10^{12}pfu/ml	3	5.67×10^{7}pfu/ml	2.44×10^{12}pfu/ml
